# Psycho-Cardiological Disease: A Bibliometric Review From 2001 to 2021

**DOI:** 10.3389/fcvm.2022.890329

**Published:** 2022-04-29

**Authors:** Yaping You, Xintian Shou, Xuesong Zhang, Shaowei Fan, Ruoning Chai, Wenjing Xue, Yuanhui Hu, Qingyong He

**Affiliations:** ^1^Department of Cardiovascular Diseases, Guang’anmen Hospital, China Academy of Chinese Medical Sciences, Beijing, China; ^2^Graduate School of Beijing University of Chinese Medicine, Beijing, China

**Keywords:** psycho-cardiological disease, cardiovascular diseases, mapping knowledge domains, cite space software, bibliometric analysis

## Abstract

The aim of this study was to gain insight into the progress and dynamics of psycho-cardiological disease research and track its hot spots. We have analyzed psycho-cardiological disease-related literature extracted from the Web of Science (WOS) Core Collection from 2001 to 2021 with the help of Cite Space. As a result, we have included 5,032 records. Then, we have analyzed connected networks for the country, author, subject category, keywords, and cited reference. We have summarized the findings in four aspects. First, the annual quantitative distribution of publications is on the rise, although there is a slight drop. Second, in terms of country analysis, the United States, England, Australia, Germany, and Italy are the main research forces in psycho-cardiological diseases. At the same time, several academic entities represented by Andrew Steptoe and Roland von Känel, MD, have been formed based on the early consciousness of physical and mental health in these countries. Besides, China is also more concerned about it due to the rapid population aging process and the largest population. Third, the psycho-cardiological disease is multidisciplinary, including psychology, psychiatry, clinical medicine, such as cardiovascular system and neurology, public environmental and occupational health, and pharmacology. Finally, the results of keyword analysis and co-cited references indicate the hot spots and frontiers in psycho-cardiological disease. The hot spots in psycho-cardiological disease include three aspects. The first aspect includes psychosocial factors, such as depression, lack of social support, and low economic and social status; the second aspect includes priority populations, such as Alzheimer’s disease dementia caregivers, elderly, and patients with cancer, and the third aspect includes interventions, such as exercise therapy and diet. In addition, there are three future research frontiers. The first is a psycho-cardiological disease in patients with COVID-19; the second is cardiac rehabilitation, especially exercise therapy and health behavior evaluation; and the final is evidence-based medical evaluation, such as systematic reviews and meta-analyses.

## Introduction

Cardiovascular diseases (CVDs) and mental illnesses have become the two major severe diseases impacting human physical and psychological health. When CVDs or symptoms and psychological disorders are present together, they are called psycho-cardiological disease (1). CVDs remain a primary reason for disability and early death worldwide (2). In disability-adjusted life years (DALYs), ischemic heart disease and stroke rank first and third, respectively, in the global burden of disease (3). In the United States, CVDs lead to health and economic burdens (4). The reported cost of treatment for CVDs in the United States in 2015 was US$213 billion (5). According to estimates, the cost of treating obesity and diabetes, both of which put people at risk for CVDs, was US$149 billion and US$237 billion per year, respectively (6, 7). In China, as issued in the official Report on Cardiovascular Diseases in 2017 by the National Center for CVDs, there were 290 million patients with CVDs (8). However, people pay less attention to the interdependence of mental health and CVDs (1). Related studies have shown a double causal link between negative emotions and CVDs.

The symptoms of CVDs may be triggered or exacerbated by negative emotions. Evidence shows that major depression was responsible for almost 4 million estimated ischemic heart disease DALYs) in 2010 globally (9). According to the data supplied, in Korea, the incidence of ischemic heart disease increased by 38% among older adults exposed to depression (10). Besides, depression is associated with increased cardiovascular adverse events, such as myocardial infarction and heart failure. In addition, recent data demonstrate that genetic risk factors for major depressive disorder (MDD) and loneliness multiply the risk of CAD in women (11). Moreover, CVDs can also contribute to psychological health problems. Depression occurs in one in five patients with coronary artery disease, peripheral artery disease, or heart failure (12). Men or women with CVDs are at high risk of stress, depression, and suicidal ideation (13). Some current research indicates that patients with heart failure are more likely to have ongoing anxiety during hospitalization and poor quality of life (14, 15). Depression and anxiety are common in atrial fibrillation and atrial flutter (16, 17).

The CVDs and mental illnesses are inextricably linked but have low clinical diagnosis rate. A retrospective analysis of 132 cases concludes that the clinical detection rate of psycho-cardiological disease was very low (1). Therefore, it is essential to pay more attention to the psycho-cardiological disease. The current distribution of literature on psycho-cardiological disease is relatively fragmented, which prevents medical practitioners interested in this field from having a comprehensive and intuitive understanding of the current status and trends of psycho-cardiological disease research in the international arena. The Cite Space software is visualization software that mainly intuitively displays the hot and cutting-edge knowledge in a specific subject field in the form of a knowledge map. There have been knowledge mapping studies of atrial fibrillation, coronary heart disease, and depression or anxiety (18, 19). Still, there have been no knowledge mapping studies at a holistic level between CVDs (e.g., coronary heart disease, atrial fibrillation, hypertension, and heart failure) and psychological diseases (e.g., depression, anxiety, and stressful trauma).

Therefore, this article visually analyzes the relevant literature on psycho-cardiological disease gained from the Web of Science (WOS) Core Collection database between January 2001 and December 2021. Based on the scientific knowledge map of psycho-cardiological disease, we have provided the research trends and hot issues in this field for related researchers.

## Materials and Methods

### Data Source

WOS Core Collection^1^ was used to obtain the publications, and the retrieval strategy was [TS = (“cardiovascular disease” AND “psychological disease” OR “Bi-heart disease” OR “psycho-cardiological disease”)] AND [language = (English)] AND [article type = (article AND reviews)] AND [Time span = (January 2001 to December 2021)]. A total of 5,032 results were included by excluding incomplete or unpublished literature, such as conference abstracts and conference papers, according to the classification tags of WOS. Later, we imported the titles, authors, abstracts, keywords, and cited references into Cite Space 5.8.R3, a scientific knowledge mapping analysis tool.

### Research Methods

After importing the literature data from the WOS data download into Cite Space 5.8.R3, deduplication was performed first. After running the deduplication program, there were no duplicates in the literature included in this study, and the original data could be used directly for subsequent analysis. The results were presented in a visual map, and nodes were the sign of analyzed items. The greater the node range, the more frequently the object appeared or the more citations there were. The thickness of the lines connecting nodes indicates the degree of co-occurrence or co-citation (20). Besides, betweenness centrality (BC), performed in purple in the knowledge map, was introduced in order to evaluate the importance of a node in the network and its value was between 0 and 1. It is generally considered that BC, with a value greater than 0.1, is a central medium (21). The bigger the size of the node, the higher the centrality. Furthermore, clustering is another way of analyzing the research domains by reflecting a unique theme. Finally, burst detection captured events’ burstness with certain features rising sharply in frequency. Therefore, the limitation of just addressing the cumulative number of metrics to measure an entity’s influence can be overcome (22).

In this study, country, author, category, keywords, and references were selected as the research items, and the time span was 2001–2021. Each year is a time slice. Links with strength set to cosine and scope set to within slices, and the 50 nodes with the highest frequency in each time period were also selected (top *N* = 50); the network using the pathfinder algorithm was pruned, and the rest of the settings were default parameters. The visualization plots and tables in stages were drawn.

## Results

### Analysis of Annual Distribution of Publication

**FIGURE 1:**
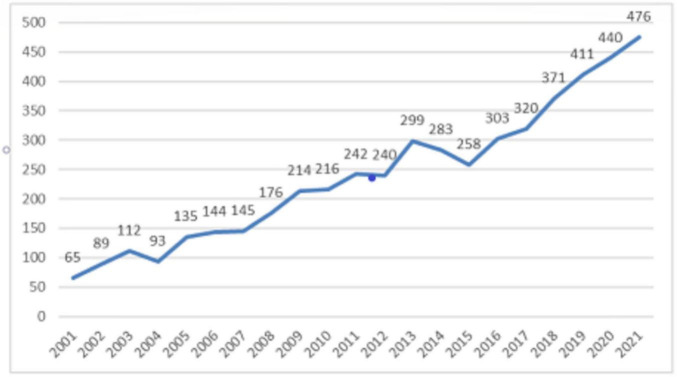
Annual quantitative distribution of the psycho-cardiological disease.

The annual quantitative distribution of publications is one of the significant signs to explain the development trend of a specific field (23), i.e., the yearly publications of research on the psycho-cardiological disease, as shown in Figure 1. The graph reveals a steady rise in the number of psycho-cardiological disease-related literature on the whole between 2001 and 2021, although there is a slight drop. As shown in Figure 1, there is an increasing trend in the amount of literature published after 2008 compared to before. In addition, it reached a small peak in 2014. In conclusion, more emphasis has been put on psycho-cardiological diseases, and an increasing number of relevant studies are underway. People are increasingly conscious of their physical and mental health.

### Analysis of Country Ranking and Co-author

Knowledge maps can provide information about significant countries and productive teams for relevant medical researchers and then can contribute to their collaboration (24). As depicted in Table 1, the United States, England, Australia, Germany, and Italy are the most influential countries for psycho-cardiological disease, not only in terms of the number of co-author papers but also in higher ranked centrality. China, with the second high degree BC, stands out in this table. A rapid aging population speed and the largest older population may have promoted the increased BC in this article (25, 26). Population aging has become a significant challenge in the world, both in developed and developing countries. Older adults often face challenges with their physical, mental, cognitive, and social health. The growing number of older adults suffering from hypertension, particularly with complications, is susceptible to coexisting depression disorders, such as depression or anxiety. In addition, 8.5–27.3% of people with diabetes suffer from depression (27). Related studies find that depression is prevalent in institutionalized older adults (28, 29). Therefore, clinicians should pay more attention to the psychological problems of older people with CVDs.

**TABLE 1:** Top 10 influent countries of the psycho-cardiological disease and relevant literature.

Rank	Country	Frequency	Burst	BC
1	United States	1,813	-	0.37
2	England	584	-	0.12
3	Australia	390	-	0.12
4	Germany	338	-	0.14
5	Italy	278	-	0.32
6	Netherlands	265	-	0.13
7	Canada	247	-	0.32
8	People’s Republic of China	213	-	0.36
9	Switzerland	206	5.13	0.14
10	Sweden	176	3.32	0.09

**FIGURE 2:**
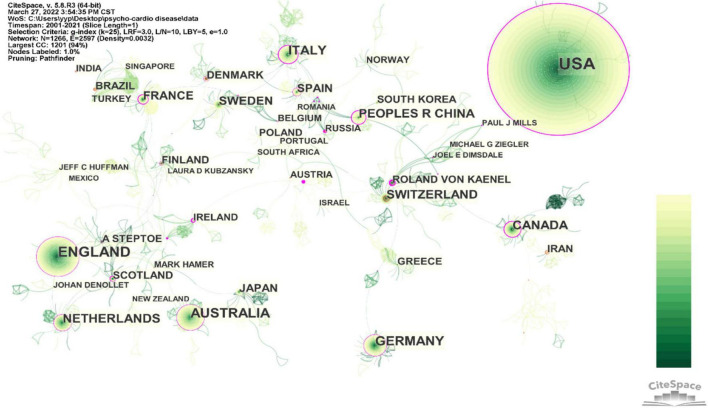
Countries–authors hybrid network of publications about the psycho-cardiological disease and related literature.

Meanwhile, several academic entities have been formed based on the early consciousness of physical and mental health in these countries (displayed in Table 2). Andrew Steptoe, Professor of Epidemiology and Public Health at UCL, believes that acute stress disorders can increase cardiac disease events (30). For example, depression, a cause of acute coronary syndrome, is also an accompanying symptom (31, 32). Roland von Känel, MD, with the highest degree of BC, a professor of Medicine and Head Division of Psychosomatic Medicine at University Hospital Bern, is a critical author in promoting the development of psycho-cardiological disease. He declares that there is an interaction between psychosocial risk factors, such as anxiety, depression, stress, and CVDs (33). Besides, he is devoted to conduct a series of clinical trials, such as on dementia caregivers with an increased inflammation marker and a higher risk of suffering from CVDs (34, 35). More importantly, he advocates the inclusion of psychosocial risk factors in cardiac rehabilitation (33). However, Figure 2 shows that the links between the nodes representing countries are more fragmented, and the connections between the nodes representing authors are stronger. Above these indicate relatively less collaboration and weak linkage between countries in psycho-cardiological disease.

**TABLE 2:** Top 10 productive authors of publications about the psycho-cardiological disease and related literature.

Rank	Author	Frequency	Burst	BC
1	A Steptoe	81	11.35	0.17
2	Roland Von Kaenel	67	17.73	0.69
3	Mark Hamer	39	6.32	0.1
4	Johan Denollet	28	4.88	0.03
5	Paul J Mills	27	9.09	0.35
6	Jeff C Huffman	27	6.5	0.07
7	Michael G Ziegler	24	7.77	0.35
8	Laura D Kubzansky	23	4.16	0.12
9	Joel E Dimsdale	23	8.72	0
10	Mika Kivimaki	21	-	0.32

### Analysis of Subject Categories’ Co-occurrence

**FIGURE 3:**
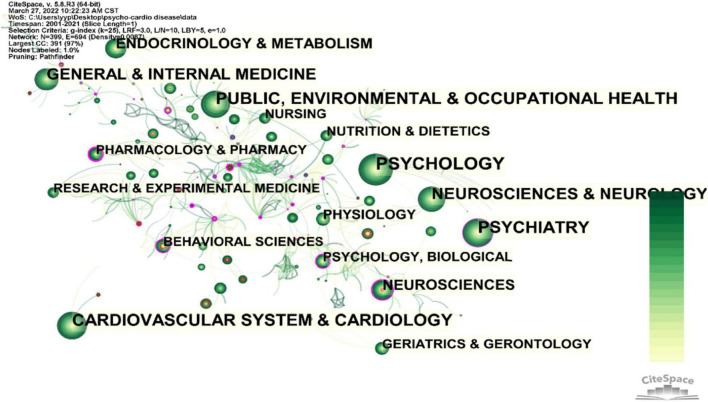
Subject category coexistence of publications about the psycho-cardiological disease and related literature.

The subject category of an article can also be regarded as evidence of a high degree of concentration in this article (22). Figure 3 expresses the subject category’s coexistence of psycho-cardiological diseases and allied research publications. As described in Figure 3, PSYCHOLOGY, PSYCHIATRY, CARDIOVASCULAR SYSTEM and CARDIOLOGY, PUBLIC, ENVIRONMENTAL and OCCUPATIONAL HEALTH, NEUROSCIENCES and NEUROLOGY, GENERAL and INTERNAL MEDICINE, ENDOCRINOLOGY and METABOLISM, and NEUROSCIENCES are noticed in Table 3. Based on these observations, we have argued that psycho-cardiological diseases’ research is multidisciplinary, belonging to the biological, psychological, and social medical model. There are varying degrees of association between bi-heart diseases and many disciplines. Psychology and clinical medicine have the highest participation, followed by public health management. The figure’s nodes of physiology, neuroscience, pharmacology, and psychiatry are marked with purple circles (BC is greater than 0.1), showing that they are worthy of attention and influential disciplines. In addition, neuroscience and behavioral sciences marked by red rings have positive burst detection, suggesting that they are popular for a certain period.

**TABLE 3:** Top 15 subject categories of publications about the psycho-cardiological disease and related literature.

Rank	Subject category	Frequency	Burst	BC
1	Psychology	924	-	0.09
2	Psychiatry	729	-	0.14
3	Cardiovascular system and cardiology	721	-	0.04
4	Public, environmental and occupational health	668	-	0.02
5	Neurosciences and neurology	528	-	0.04
6	General and internal medicine	486	-	0.04
7	Endocrinology and metabolism	296	-	0.05
8	Neurosciences	280	5.65	0.21
9	Physiology	165	-	0.23
10	Psychology, biological	155	-	0.11
11	Geriatrics and gerontology	151	-	0.03
12	Nutrition and dietetics	149	-	0.05
13	Pharmacology and pharmacy	145	-	0.68
14	Research and experimental medicine	142	-	0.01
15	Behavioral sciences	140	4.89	0.02

The first burst detection is neuroscience. Perhaps, because the population in the world is aging rapidly, the number of older adults with Alzheimer’s dementia is gradually increasing. Evidence shows that there will be more than 13.8 million Americans aged 65 years and older with Alzheimer’s disease by midcentury. Moreover, official death evidence announces that Alzheimer’s disease is the sixth leading cause of death in the United States (36). As a result, the burden of caring for this group has also increased. A study reports that dementia caregivers had a higher prevalence of depression (30–40%) than other caregivers, such as those who helped people with schizophrenia (20%) or patients with stroke (19%), with an increased risk of CVDs (37). In a similar case, relevant research suggests that the symptoms of depression are associated with interleukin-6 and D-dimer and have increased the risk of CVDs in caregivers (38, 39).

The second burst detection is behavioral sciences. Because of the increasing number of people with Alzheimer’s dementia, there is a growing interest in behavioral activation and an effective way to reduce depression and improve the quality of life of caregivers of people with dementia (40). Moore applies pleasant events program (PEP) therapy and homework therapy to divide randomly 100 dementia family caregivers into a PEP intervention group and an information support control group. The results show that behavioral activation can effectively lower interleukin-6 in caregivers and can reduce the risk of CVD in caregivers (41). In addition, a longitudinal study of the effects of dementia care stress on caregivers’ health by Von Känel expresses that family caregivers of patients with dementia had higher plasma interleukin-6 and D-dimer levels, which in turn led to an increased risk of CVDs (35).

### Analysis of Keywords

**FIGURE 4:**
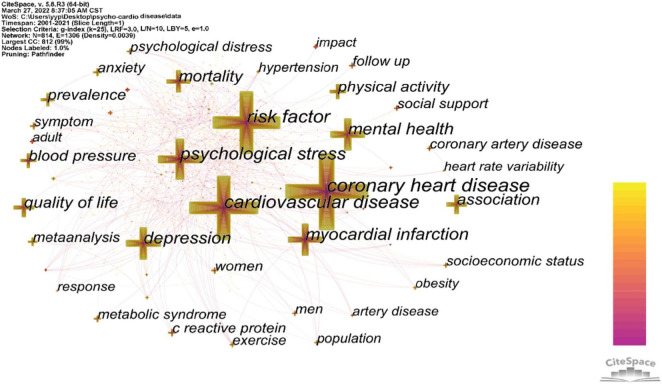
Network of the keywords in publications about the psycho-cardiological disease and related literature.

Investigating keywords can provide a richer explanation in understanding the concentration of research topics for medical researchers (22). Since keywords explain the central ideas of the article, the content of the article is summarized and condensed, and the content of the literature is mirrored. Based on the literature content, Cite Space extracts the article’s keywords and analyzes them to explore and discover the hot issues in this research field. Figure 4 exhibits keywords’ coexistence. The keywords were expressed in the form of cross. The larger the size of the cross, the more often the keywords appear. In fact, the greater the number of keywords, the harder it is to analyze (42). Cluster analysis can contribute to overcome this limitation (22).

**FIGURE 5:**
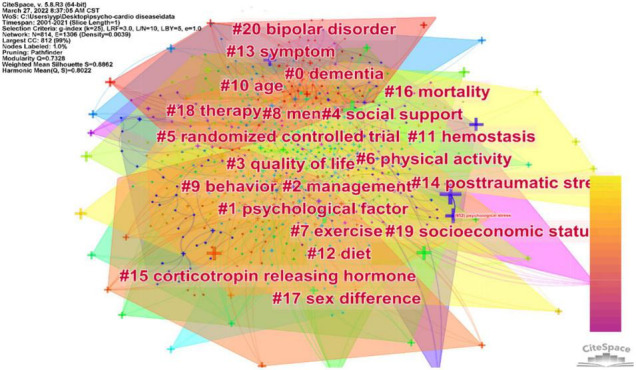
Network of the keyword clusters in publications about the psycho-cardiological disease and related literature.

Figure 5 describes the clustering of 21 keywords based on the log-likelihood ratio (LLR) algorithm. To further refine the research content and better grasp the research hot spots, 21 clusters are divided into 6 parts, including target population (#0dementia), psychosocial risk factors (#1psychological factor, #4social support, #8men, #10age, #14posttraumatic stress, #19 socioeconomic status, #20bipolar disorder, and #17sex difference), intervention (#3quality of life and #16mortality), clinical trials (#5randomized controlled trial), pathogenesis (#11hemostasis and #15 corticotropin releasing hormone), and clinical manifestations (#13symptom).

First, it is worth mentioning the psychosocial risk factors in bi-heart diseases. Psychosocial risk factors mainly contain lack of social support (#4), low economic and social status (#19), stress at work and family, bipolar disorder (#20), posttraumatic stress disorder (#14), and psychological factor (#1), such as depression, anxiety, anger or hostility, gender differences (#17), and personality factors. All of the above risk factors increase the risk of CVDs (43–45). For example, depression is coupled with increased body mass index and obesity, an independent risk factor for CVDs (46, 47). In support of this opinion, Johnson and colleagues assessed patients’ mental health who lived with spontaneous coronary dissection and found significantly higher depression, anxiety, and posttraumatic stress disorder (48). In addition, reported data suggest that in epidemiology, women may have a larger share of depression-related CVDs, which is partly attributable to genetic factors (49).

Second, bi-heart disease aims to improve health-related quality of life and reduce mortality (#16). The main treatment measures (#18) include exercise therapy (#6, #7) and risk factor management (#12), which are effective clinical practices recommended by the U.S. Preventive Services Task Force to reduce CVD risk among high-risk individuals (50). Besides, these interventions are among the top five prescriptions for cardiac rehabilitation (51).

Third, in mechanism, it links inextricably to the imbalance of the hypothalamic–pituitary–adrenal axis (#15) and platelet activation (#11). Studies show that long-term chronic stress, such as depression and anxiety, can lead to hyperfunction of the HPA axis and excessive secretion of cortisol. In addition, it can also cause hyperlipidemia and hypertension and then leads to various CVDs (52). Chronic stress leads to platelet aggregation and contributes to develop CVDs, suggesting that platelet aggregation may increase the incidence of cardiovascular adverse events (53). The focus population in bi-heart disease is people caring for people with dementia, who are at higher risk for CVDs and should be paid attention to (27).

Finally, the field of bi-heart disease attaches great importance to clinically randomized controlled trials, which rank second in the evidence pyramid proposed by the Medical Center of State University of New York in 2001. In addition, it is in line with evidence-based medicine-level recommendations.

### Analysis of Reference Co-citation

**FIGURE 6:**
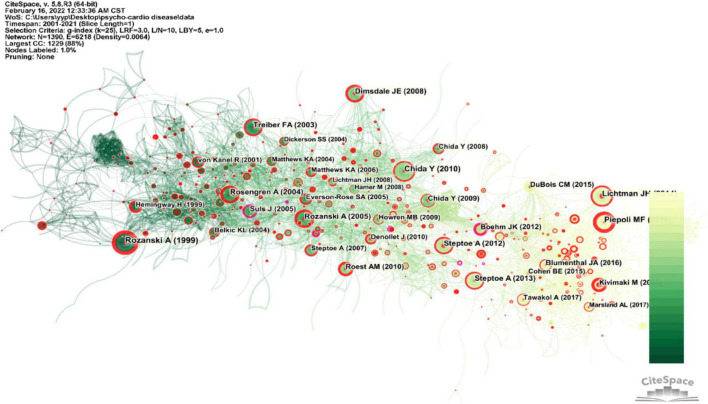
Co-citation network of references cited by publications about the psycho-cardiological disease and related literature.

Co-citation analysis of documents refers to two or more papers being cited in the same document (54). Each node represents a cited article, and the node’s size reflects the proportion of citations it has received. Citations with citation bursts are shown in red rings, while nodes in purple rings have higher betweenness center values. By analyzing the clusters and key nodes in the collaborative citation network, the knowledge structure of the research field and its changes can be revealed. Figure 5 shows a co-citation network of bi-heart disease and related research references. As displayed in Figure 6, a circular node represents a reference, and the larger the node’s size, the more frequently the reference is cited. The purple-circled nodes in the network (BC is greater than 0.1) illustrate that they are pivotal turning points driving the field of bi-heart disease.

The first turning point occurred in 2005. Before 2005, all the studies were on the relationship between adverse psychological factors, such as anxiety, depression, and hostility and health. However, after 2005, increasing evidence showed a protective association between positive psychological factors and physical health. Focusing on positive psychological wellbeing (PPWB) is associated with the most cardiovascular-related health behaviors [e.g., adequate sleep, fruit and vegetable intake, and biological mechanisms (inflammation and metabolic processes)] (55). It is consistent with the ideal cardiovascular health proposed by the American Heart Association in 2010. The second turning point occurred in 2010, i.e., a randomized clinical trial of cognitive-behavioral therapy for recurrent CVDs and recurrent myocardial infarction. The results suggest that cognitive-behavioral therapy can reduce the risk of recurrent CVDs and myocardial infarction compared with traditional treatment methods and can be included in the secondary prevention program for coronary heart disease (56). The third turning point occurred in 2012. As Fang, MD, noted that newly diagnosed cancer was traumatic stress, conducting a historical cohort study showed that cancer increased the patient’s suicide rate and the risk of cardiovascular events (57). All in all, these three turning points are virtual nodes to move the development of the field of bi-heart disease.

**FIGURE 7:**
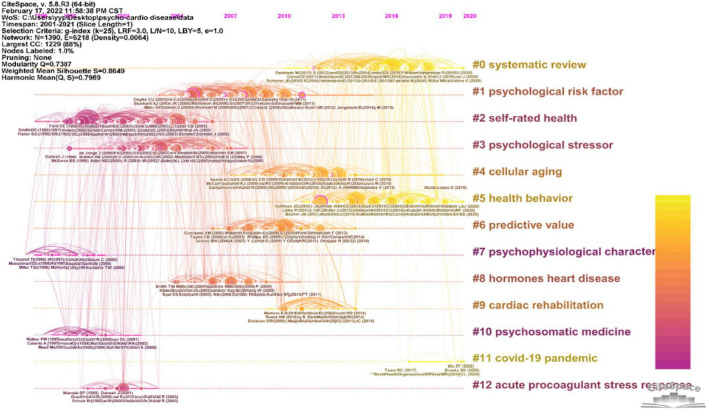
Timeline view of references cited by publications about the psycho-cardiological disease and related literature.

Furthermore, Cite Space can also display hot knowledge on bi-heart disease in a timeline view (shown in Figure 7). The network consists of highly cited references within a given slice of time. Nodes are sorted in the same lines by a clustering technique called brilliant local moving. Clusters are numbered so that groups containing more references are ranked higher (22). The color legend at the top shows that cooler-colored links and citations occurred in 2001, while hotter links and citations occurred closer to 2021. We can track the topic trend and time development research on psycho-cardiological diseases by referring to the legend. According to the timeline view, we can assume that the emphasis on studies related to the early bi-heart disease mainly focuses on drug treatment (#10), disease-related self-health evaluation (#2), psychological factors (#1 and #3), and psychophysiological characteristics (#7). Midterm gradually shifted to pathogenesis (#4, #12), disease predictors (#6), and hormones’ heart disease (#8). Later, it also focuses on the evaluation method of the disease (#0), personal health behavior (#5), cardiac rehabilitation (#9), and the new COVID-19 pandemic (#11).

The first to note is the COVID-19 pandemic (#11). COVID-19 pandemic has swept the world, posing a threat to human health. Studies have shown that COVID-19 can lead to overwhelming stress, addiction to tobacco, alcohol, or increased obesity. “COVID-19 related depression” reflects the condition (58). Pressure from the coronavirus can lead to stress cardiomyopathy or an increased risk of heart failure (59). It reminds scientists and doctors worldwide to pay attention to the psychological-heart disease caused by the new coronary pneumonia virus.

The second one is cardiac rehabilitation (#9). From the simple drug treatment (#10) at the beginning, the treatment of bi-heart disease has gradually emphasized the importance of cardiac rehabilitation. In addition to treating the disease itself, it is also necessary to focus on the patient’s mental health and the ability to reintegrate into society. Studies have shown that cardiac rehabilitation has a good effect on chronic heart failure (60). In cardiac rehabilitation, there are five prescriptions, including drugs, exercise, nutrition, psychology (e.g., sleep management), and CVD risk factor management (e.g., smoking, alcohol, blood pressure, and obesity) (61). The most important core content is exercise. Traditional Chinese medicine exercises play a significant role in cardiac rehabilitation. There is evidence that tai chi improves cardiac function, quality of life, and anxiety and depression in patients with coronary artery disease (62). In addition, a randomized controlled trial shows the tai chi group had significantly lower blood pressure, lower blood glucose index, exercise self-efficacy, and perceived stress, and improved mental health compared to the brisk walking group with hypertension (63). In addition, healthy behavior (#5) is one of the quality evaluations of cardiac rehabilitation development. During the COVID-19 pandemic, cardiac rehabilitation is one of the effective treatments for patients with new coronary pneumonia complicated by psycho-cardiological disease (64).

Third, the current literature evaluation in psycho-cardiological disease is mainly a systematic review. The systematic review (#0) and meta-analysis are at the top of the 9th piece of evidence in the American pyramid, indicating that the research on bi-heart disease is consistent with evidence-based medical research, and the conclusion is reliable. At present, systematic review and meta-analysis are the more commonly used evaluation methods in psycho-cardiological disease (65–68).

## Discussion

With the establishment of the biopsychosocial medicine model, medicine gradually shifted from disease-centered to integrated mind-body medicine, so psycho-cardiology was born, and psycho-cardiological disease was proposed. The forms of psycho-cardiological illness are divided into three types. The first type is psychological, emotional, and mental problems caused by symptoms similar to CVDs, such as chest tightness, chest pain, and palpitations. The second type is CVD combined with psychological issues. When the diagnosis is clear (coronary artery disease or heart failure connected with depression or anxiety), clinicians can quickly prescribe the proper medication. When the diagnosis is not precise (mostly arrhythmias), patients may also show negative psychological or emotional symptoms, such as anxiety and depression. For example, Brugada syndrome, long QT syndrome, hypertension, hyperthyroidism, and lifestyle factors, such as regular endurance exercise, can contribute to atrial fibrillation, affecting the patient’s quality of life and leading to potential initial depression (69–72).

The final form is a heart disease that undergoes certain stressful blows that cause psychological changes. Today some medical approaches may impact the quality of life or health of the patient. A recent study has shown that catheter ablation makes asymptomatic atrial fibrillation patients more health-conscious, which leads to better medication adherence, lower salt-sensitive hypertension, and thus improved cardiac function. It also reduced mental anxiety and activity limits, which helped improve the quality of life (73). However, another study suggests that anxiety and depression in patients with the acute coronary syndrome who underwent PCI were associated with increased length of hospital stay (74). Therefore, medical interventions have a two-sided impact on the patient’s quality of life and health, and clinicians should assess the patient’s psychological status.

In this review, an analysis of the literature in this article shows an overall upward trend in research on psycho-cardiological disease from 2001 to 2021, indicating an increasing interest in physical and mental disorders and the importance of psychological factors in disease. From the visual map of countries and authors, countries, such as the United States, the United Kingdom, Australia, and Italy, are the main research forces in bi-heart disease. It shows that the countries mentioned above have a high scientific output in this field, which helps relevant researchers to seek collaboration. In addition, several academic groups have formed, such as the Andrew Steptoe and Roland von Känel, MD, as the core academic group. However, research in this area is fragmented, and there is a lack of cooperation and anxiety between countries. Hence, it is necessary to strengthen international communication and collaboration in this field and form an interactive global cooperation network to promote in-depth research.

As can be seen from the subject categories, the field of bi-heart disease is a typical interdisciplinary subject, including clinical medicine and psychology, psychiatry, public health and occupational health, nursing, and pharmacology, which suggests that a growing number of people pay attention to bi-heart disease, not only in the screening and diagnosis of the disease but also in the treatment and care of the disease. Correspondingly, it is necessary to cultivate high-level compound medical talents. At the same time, a multidisciplinary collaborative disease management team should be formed, which is beneficial for the individualized and precise treatment of psycho-cardiological disease.

A visual map of keywords and co-cited references reflects hot spots and trends in bi-heart disease. First, from the keyword network mapping in this article, we can observe that coronary artery disease or myocardial infarction combined with anxiety and depression is more common. In contrast, atrial fibrillation combined with anxiety and depression accounts for a smaller proportion. It may be because the diagnosis of atrial fibrillation is relatively unclear. Second, the early bi-heart disease field focuses on the association between psychological factors and CVDs and the mechanisms. In addition, there are three hot spots of research on psychosocial risk factors, priority populations, including Alzheimer’s disease dementia caregivers, patients with cancer, and elderly, and interventions, such as exercise and diet. It is worth being concerned about the exercise therapy. Regular exercise can improve the patient’s cardiovascular symptoms and psychological status, while prolonged endurance exercise can lead to adverse cardiovascular events, such as atrial fibrillation (72). Therefore, low-to-moderate intensity exercises, such as tai chi and Baduanjin, in cardiac rehabilitation are recommended.

Third, the frontiers of current research are evaluation methods, such as systematic reviews and meta-analyses, COVID-19 populations, cardiac rehabilitation, and healthy behaviors. Finally, three turning points must be mentioned, the first in 2005 focusing on positive psychological factors being protective against CVD, the second in 2010 with a focus on cognitive-behavioral therapy, and the third in 2012. The turning point is that patients with cancer are susceptible to stress cardiomyopathy.

## Limitation

A limitation of this study is that it only uses the WOS database. It is mainly the limitation of the Cite Space software itself. The citation and quoted information in scientific texts reflect the flow and dissemination of scientific knowledge, an essential theoretical basis for knowledge mapping. Most of the current visualization tools are developed based on WOS. WOS is the most representative and widely used in this field, so it is used. Besides, Cite Space software can only analyze a single database. For these reasons, the literature search in this study was not comprehensive, which may cause biased results.

## Conclusion

People are increasingly concerned about the harmony and health of the mind and body. Research in psycho-cardiological disease focuses on psychosocial factors, priority populations, such as Alzheimer’s disease dementia caregivers, elderly, and patients with cancer, and interventions, such as exercise therapy. In addition, there are three future research frontiers. One is psychological heart disease in patients with COVID-19, the second is cardiac rehabilitation, especially exercise treatment and health behavior evaluation, and the final one is an evidence-based medical evaluation.

## Author Contributions

YH, QH, and YY designed the research plan and revised the manuscript. YY, XS, and XZ wrote the manuscript. SF, RC, and WX were responsible for the use of software and document retrieval. All authors contributed to the article and approved the submitted version.

## Conflict of Interest

The authors declare that the research was conducted in the absence of any commercial or financial relationships that could be construed as a potential conflict of interest.

## Publisher’s Note

All claims expressed in this article are solely those of the authors and do not necessarily represent those of their affiliated organizations, or those of the publisher, the editors and the reviewers. Any product that may be evaluated in this article, or claim that may be made by its manufacturer, is not guaranteed or endorsed by the publisher.

## Footnotes

1
http://apps.webofknowledge.com
